# Not All Activities Are Equal: Diverse Physical Activities and Well-Being in Indian Older Adults – LASI Findings

**DOI:** 10.1093/geroni/igaf122.2746

**Published:** 2025-12-31

**Authors:** Urmimala Ghose

**Affiliations:** Humboldt University in Berlin, Humboldt University in Berlin, Berlin, Germany

## Abstract

Engagement in physical activity is widely recognized as a key factor in promoting well-being in later life. However, most research on this relationship has been conducted in Western contexts, with limited attention to diverse forms of physical activity in non-Western populations. This study examines the association between different intensities of physical activity—vigorous, moderate, and mild physical activities (e.g., Yoga, Pranayama)—and well-being outcomes, including life satisfaction and depressive symptoms, among older adults in India. Using data from the Longitudinal Aging Survey of India (LASI; N = 73,396, Mean age = 57.92, women = 57.58%, 35.96% having at least secondary education, 76.69% married), we conducted multiple regression analyses to assess both direct effects and moderation by age, gender, and health status. Results indicated that moderate physical activities were consistently associated with higher life satisfaction and lower depressive symptoms, whereas mild physical activities showed mixed effects depending on health status. Vigorous activities, while significant, had weaker and sometimes negative associations with well-being. Moderation analyses revealed that the benefits of physical activities varied by age, gender, and health, suggesting that a one-size-fits-all approach may not be optimal. These findings underscore the need to consider the diversity of physical activities in aging research, particularly in non-Western populations, to better understand how different activity intensities contribute to well-being. Tailoring physical activity recommendations to culturally relevant practices and individual health conditions may improve engagement and maximize psychological benefits for older adults.

